# Complete mitochondrial genome of the mantis shrimp *Taku spinosocarinatus* (Fukuda, 1909) (Stomatopoda: Gonodactyloidea: Takuidae) in South Korea

**DOI:** 10.1080/23802359.2020.1831994

**Published:** 2020-10-27

**Authors:** Hee-seung Hwang, Jiyeong Shin, Jongwoo Jung

**Affiliations:** a Research Institute of EcoScience, Ewha Womans University, Seoul, Korea; b The Division of EcoCreative, Ewha Womans University, Seoul, Korea; c Department of Science Education, Ewha Womans University, Seoul, Korea

**Keywords:** Crustacea, mitochondrial DNA, mitochondrial genome, stomatopoda, *Taku spinosocariantus*

## Abstract

The superfamily Gonodactyloidea is polyphyletic because of Hemisquillidae, but to date, mitochondrial genome of that family does not exist. As valuable data that can be compared in the future with that family within this superfamily, we report the first complete mitochondrial genome sequence of *Taku spinosocarinatus* of the family Takuidae. The mitochondrial genome is 15,960 bp in length and consists of 13 protein-coding genes (PCGs), 22 transfer *RNA* genes, 2 ribosomal *RNA* genes, and a non-coding A + T rich region. The overall base composition in the heavy strand was as follows: A: 34.2%, G: 13.4%, C: 19.8%, and T: 32.6%, with a G + C content of 33.2%. Phylogenetic analysis revealed that this species was most closely related to *Gonodactylus chiragra* of Gonodactylidae, registered with NCBI to date. The result of this study will enable additional comparisons between families in the future.

The crustacean order Stomatopoda consists of seven superfamilies and they have large and powerful raptorial appendages that can be used for ‘spearing’ or ‘smashing’ (Caldwell and Dingle 1976). Among them, Gonodactyloids comprise seven families and most have a smashing-type raptorial claw. According to stomatopod molecular phylogenetic studies, this superfamily is polyphyletic (Ahyong 1997; Hof 1998; Ahyong and Harling 2000; Wal et al. 2017) because of the family Hemisquillidae. The present species, *Taku spinosocarinatus* belongs to Takuidae within Gonodactyloidea has a smashing-type of raptorial claw like Hemisquillids. Since previous study (Wal et al. 2017) showed that smashing group in other superfamily had a monophyletic origin, the case of these families is an exception. Unfortunately, studies on complete mitochondrial genome have not progressed. Aiming to be able to comparable with the family Hemisquillidae and improve the phylogenetic knowledge on these families within this superfamily, and further characterize their mitochondrial gene order by sequencing the mitochondrial genome of *Taku spinosocarinatus*.

The specimen was collected by scuba diving in the subtidal zone of Dokdo Island, South Korea (geographic location: 37°14′34.9″N 131°52′08.6″E) on 1 June 2019, and was preserved in 95% ethyl alcohol. The specimen was deposited at the Research Institute of EcoScience, Ewha Womans University (EWNHMAR768). Total DNA was extracted from leg muscle tissue using DNeasy Blood and Tissue kit (Qiagen, Hilden, Germany) and the DNA library was prepared using TruseqNano DNA Prep Kit (Illumina, San Diego, CA, USA). The mitochondrial DNA was sequenced using Illumina Novaseq 6000 and MITObim (Hahn et al. 2013) was used for the assembly of the complete mitochondrial genome, which was annotated using MITOS (Bernt et al. 2013).

The mitochondrial genome comprised a total of 15,960 bp, which encoded 13 proteins, 22 transfer RNAs, two ribosomal RNAs, and a putative control region. For the protein-coding genes (PCGs), the most common shared start codon was ATG identified in *COX2*, *COX3*, *NAD3*, *NAD4*, *NAD5*, *NAD4L*, *ATP6*, and *CYB*. The starting codon used by *COX1* was ACG, which is often observed in the *COX1* gene of malacostracan mtDNAs (Cook 2005; Liu and Cui 2010), whereas for *NAD1* was ATA, for *ATP8* was ATC, and for *NAD2* and *NAD6* was ATT. The most frequent stop codon was TAA, except for *COX2*, *NAD1*, and *NAD6* that had AAT, TAG, and CCT as stop codons. In particular, NAD1 and NAD6 proteins ended with an incomplete stop codon. Such cases have been identified in several PCGs of all stomatopod mitochondrial genomes published to date, which is attributed to excessive polyadenylation (Ojala et al. 1980, 1981). The overall mitochondrial base composition of this genome was A: 34.2%, T: 32.6%, G: 13.4%, and C: 19.8%, with a G + C content of 33.2%. The length of *LrRNA* and *SrRNA* genes in this species was 1367 and 837 bp, respectively., while the length of the transfer RNAs identified ranged from 65 to 74 nucleotides. The putative control region comprised 1069 bp and was located after transfer RNA-Val and *SrRNA*.

To explore the phylogenetic position of *T. spinosocarinatus*, we investigated the molecular phylogenetic relationships among stomatopod species using the complete mitochondrial genome sequence of seven species (Figure 1). The phylogenetic tree was constructed based on sequences of 13 PCGs identified by the maximum likelihood (ML) method using MEGA X (Kumar et al. 2018). The GTR + G + I model was identified as the best-fit model for the data, using ModelFinder (Kalyaanamoorthy et al. 2017) with 1000 bootstrap replicates.

**Figure 1.**
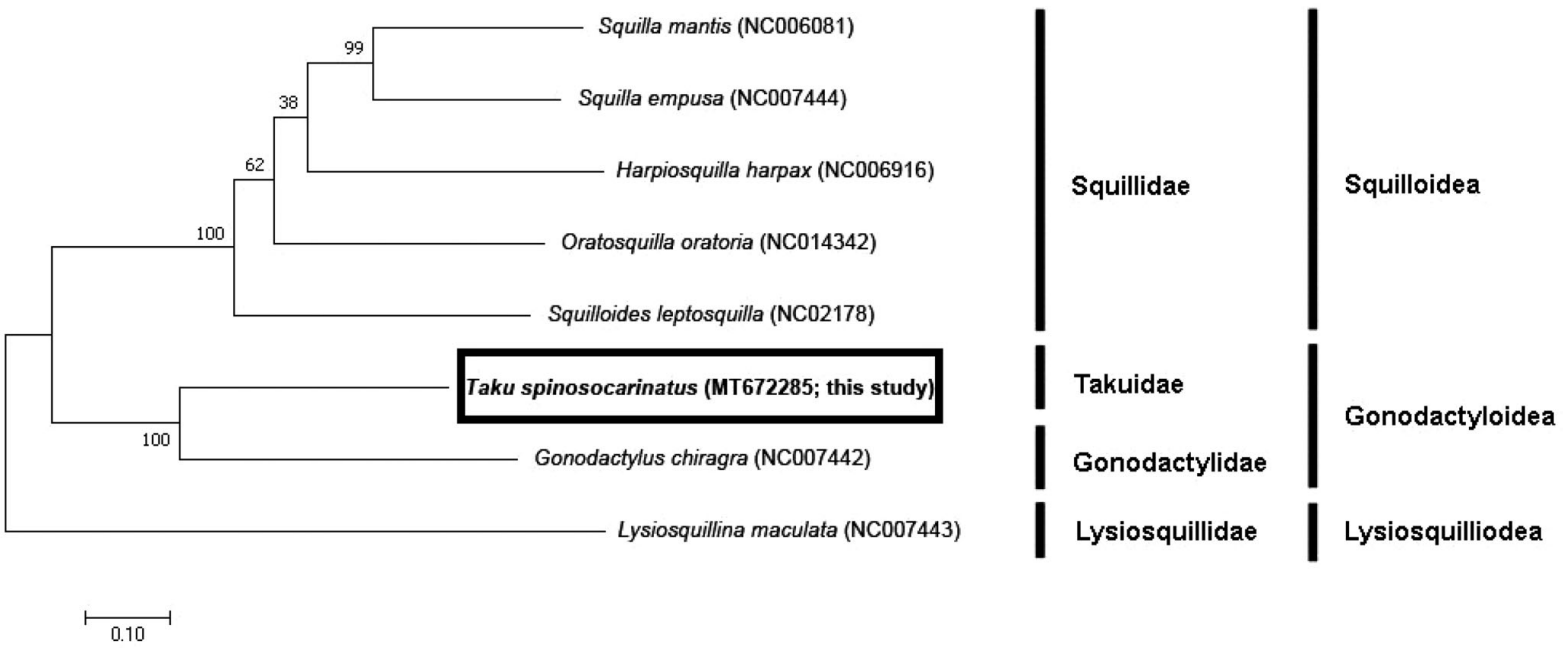
Phylogenetic tree of complete mitochondrial genomes from eight stomatopods (*Oratosquilla oratoria* (NC014342), *Gonodactylus chiragra* (NC007442), *Harpiosquilla harpax* (NC006916), *Squilla empusa* (NC007444), *Squilla mantis* (NC006081) *Lysiosquillina maculata* (NC007443), and *Taku spinosocarinatus* (MT672285)) constructed using maximum likelihood (ML) method.

One gonodactyloid species and five squilloid species were used, with a lysiosquilloid species as an outgroup. Phylogenetic analysis revealed that this species was most closely related to *Gonodactylus chiragra* of Gonodactylidae, which is a smashing group within Gonodactyloidea and registered with NCBI to date. As this is the first record of the complete mitogenome sequence of the family Takuidae, it will enable further comparisons between families for future studies about this superfamily.

## Data Availability

The genome sequence data that support the findings of this study are openly available in GenBank of NCBI at https://www.ncbi.nlm.nih.gov under the accession no. MT672285. The associated BioProject, SRA, and Bio-Sample numbers are PRJNA661984, SRR12640676, and SAMN16072594, respectively. The data that support the findings of this study are also openly available in Mendeley Data at http://dx.doi.org/10.17632/kg3ypr2cww.1
